# Prenatal Detection of Congenital Heart Diseases: One-Year Survey Performing a Screening Protocol in a Single Reference Center in Brazil

**DOI:** 10.1155/2014/175635

**Published:** 2014-01-12

**Authors:** Luciane Alves Rocha, Edward Araujo Júnior, Liliam Cristine Rolo, Fernanda Silveira Bello Barros, Karina Peres da Silva, Ana Teresa Figueiredo Stochero Leslie, Luciano Marcondes Machado Nardozza, Antonio Fernandes Moron

**Affiliations:** ^1^Fetal Cardiology Unit, Department of Obstetrics, Federal University of São Paulo (UNIFESP), 05303-000 São Paulo, SP, Brazil; ^2^Discipline of Neonatology, Department of Pediatrics, Federal University of São Paulo (UNIFESP), 05303-000 São Paulo, SP, Brazil

## Abstract

*Objective*. To describe the experience of a tertiary center in Brazil to which patients are referred whose fetuses are at increased risk for congenital heart diseases (CHDs). *Methods*. This was a cross-sectional observational study. The data was collected prospectively, during the year 2012, through a screening protocol of the fetal heart adapted from the International Society of Ultrasound in Obstetrics and Gynecology (ISUOG) guideline. We performed a fetal echocardiogram screening for all pregnant women who were referred to the fetal cardiology outpatient obstetrics clinic of a university hospital. The exams were classified as normal or abnormal. The cases considered abnormal were undergone to a postnatal echocardiogram. We categorized the abnormal fetal heart according to severity in “complex,” “significant,” “minor,” and “others.” *Results*. We performed 271 fetal heart screening. The incidence of abnormal screenings was 9.96% (27 fetuses). The structural CHD when categorized due to severity showed 48.1% (*n* = 13) of “complex” cases, 18.5% (*n* = 5) “significant” cases, and 7.4% (*n* = 2) “minor” cases. The most common referral reason was by maternal causes (67%) followed by fetal causes (33%). The main referral indication was maternal metabolic disease (30%), but there was just one fetus with CHD in such cases (1.2%). CHDs were found in 19/29 fetuses with suspicion of some cardiac abnormality by obstetrician (65.5%). *Conclusion*. We observed a high rate of CHD in our population. We also found that there was higher incidence of complex cases.

## 1. Introduction

Congenital heart diseases (CHDs) are the most common abnormalities in fetuses, being six times more common than chromosomal abnormalities and four times more common than neural tube defects [1]. The incidence of CHD with intrauterine diagnosis ranges from 2.4% to 54% [2–7]. Some countries have high incidence of CHD because they have instituted an organized policy to perform heart screening by ultrasound systematically [8–10].

A detail evaluation of the fetal heart optimizes the diagnosis of CHD [11]. This provides an appropriate prenatal and postnatal planning, enabling an improvement in neonatal morbidity and surgical outcome [1, 12–15]. Therefore, there is an increasing interest in improving detection of the cardiac defects.

There are many epidemiological and ultrasonographic data reported [2–7]; however, to the best of our knowledge, there are no published Brazilian epidemiological data. Our aim is to describe the experience of a tertiary center in Brazil to which patients are referred whose fetuses are at increased risk for CHD. After knowing the epidemiological features of our population, we may improve the future screening and treatment of CHD.

## 2. Methods

This was a cross-sectional observational study. The data was collected prospectively during the year of 2012, by a screening protocol of the fetal heart adapted from the International Society of Ultrasound in Obstetrics and Gynecology (ISUOG) guideline [10, 16].

The study population was pregnant women who were referred for prenatal assessment for suspicion or with some risk of CHD. The exams were performed in the Fetal Cardiology Unit, Department of Obstetrics, Federal University of São Paulo (UNIFESP), which is a tertiary referral center in Brazil. We recorded the indications of fetal heart screening, maternal and gestational age, fetal heart screening findings, and extracardiac abnormalities.

Fetal hearts were examined by two-dimensional, pulsed, wave and color Doppler echocardiographic methods using the Voluson E8 machine (General Electric, Medical System, Zipf, Austria) equipped with a convex transducer (RAB 4-8L). All exams included a two-dimensional evaluation of cardiac structures with the “basic” (four-chamber view of the fetal heart) and the “extended basic” cardiac screening examination (views of the outflow tracts) [10, 16]. We also performed the ductal and aortic arches position and we used the color Doppler. We assessed the cardiac situs, rhythm, venous inflow, atrial and ventricular chambers, atrioventricular and semilunar valves, and ventriculoarterial connections [10, 16].

According to our protocol, the exams were classified as “normal” or “abnormal.” The cases considered “abnormal” were undergone to a postnatal echocardiogram at the same hospital. We categorized the abnormal fetal heart according to complexity of the heart anatomical abnormalities in “complex,” “significant,” “minor,” and “others” (Table 1) [17, 18].

The data were entered into a specific protocol and were transferred to a spreadsheet within the Excel 2007 software (Microsoft Corp., Redmond, WA, USA). The statistical analysis was realized using the Stata software version 12.1 (StataCorp LP, College Station, TX, USA). We performed Chi Pearson and Exact Fisher tests for categorized variable and Mann-Whitney test for quantitative variable. We used the significance level of *P* < 0.05.

## 3. Results

We performed heart screening in 271 fetuses during a period of one year, of which, 27 fetuses had CHD (9.96%). All patients were similar except for indication of screening (Table 2), because the most common referral reason was by maternal causes (67%) followed by fetal causes (33%). Maternal causes for referral were advanced maternal age, preexisting metabolic disease, exposure to teratogens rate, maternal infection, and family history of CHDs. Fetal causes for referral were abnormal sonographic findings during routine assessment (increased nuchal translucency thickness, extracardiac defects, or suspicion of cardiac abnormalities) (Table 3).

The main referral indication was maternal metabolic disease (30%), but there was just one fetus with CHD in such cases (1.2%). CHDs were found in 19/29 fetuses with suspicion of some cardiac abnormality by obstetrician (65.5%). Then, referral indications for fetal heart screening were appropriate in cases where obstetricians suspected CHD (Table 3).

**Figure 1:**
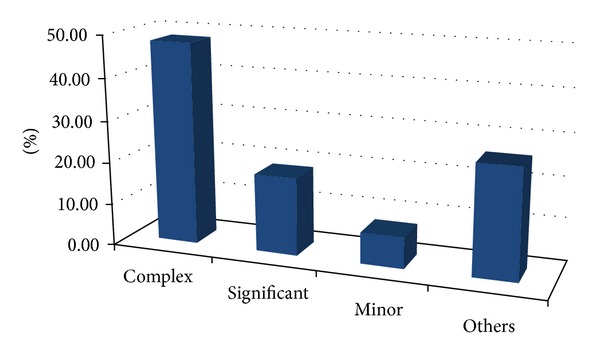
Congenital heart disease categorized by severity: complex, significant, minor, and others.

We identified 48.1% (*n* = 13) complex cases, 18.5% (*n* = 5) significant cases, 7.4% (*n* = 2) minor cardiac anomalies, and 26% (*n* = 7) others. Others cases were dysrhythmia (complete atrioventricular dissociation), hypertrophy myocardial, dextroposition secondary, and ductus arteriosus restrictive (Figure 1 and Table 4).

All CHD cases with prenatal diagnosis were submitted to a postnatal echocardiogram at the same hospital to testify the diagnosis. The mortality in one month was high (47.3%), probably because we had many complex and significant cases.

## 4. Discussion

This study showed that the incidence of CHD in fetuses (9.9%) corroborates with the findings of the literature [2–7, 9] and this is our major contribution. The prenatal incidence of CHD has a great variability ranging from 2.4% to 54%. This variability depends on the performing of a systematic screening in each country. Published Brazilian epidemiological data consider just the prevalence of CHDs in children [19–21] and lack any data of prenatal incidence.

Regarding the referral indications for fetal heart screening, the maternal metabolic disease was greater than all the other risk factors (30%). This is in discordance with the literature that already reported a greater indication of fetal heart screening for increased nuchal translucency [6], intrauterine fetal death in previous pregnancy [3], finding abnormal prenatal sonographic [22, 23], and family history of a child with CHD [24].

We observed that the indications for fetal heart screening were appropriate in cases where obstetricians suspected CHD (65.5% with CHD). This demonstrates that our obstetricians are accomplishing a good evaluation of the fetal heart; however, we must consider that this study was conducted in a university hospital in our country and it does not portray the reality of all obstetricians.

The complex and significant cases were more common among CHD (66.6%). This finding suggests that in general our cases are very severe which can justify the high mortality in our center. There are other researches about incidence of CDH that show the same severity of heart disease [23, 24] and its related to fatal cases.

We have some limitations regarding this study. First, we had an absolute small number of abnormal cases in our cohort which can prevent a more detailed statistic analyze. Second, as this work was accomplished in a tertiary center, it is difficult to generalize our data to other centers. However, it can show a picture of the incidence of CHDs when the screening is performed in a systematic way.

We observed a high rate of fetal heart disease in our population. We also found, as expected, that there was a higher incidence of complex cases. We recommend that continuous efforts should be made for prenatal screening program for CHD. We believe that with the knowledge of these data we can improve the outcomes of morbidity and mortality of children in our institution.

## Figures and Tables

**Table 1:** Classification system of fetal heart diseases used according to complexity of the heart anatomical abnormalities.

Classification	Fetal heart diseases
Complex	Heterotaxy or atrial isomerism, atresia or severe hypoplasia of a valve or chamber (hypoplastic left heart syndrome, pulmonary atresia, tricuspid atresia, aortic atresia, mitral atresia, and Ebstein's anomaly), and abnormalities of the valve inlet or outlet (complete atrioventricular septal defect, truncus arteriosus, double inlet left or right ventricle, and double outlet left or right ventricle congenitally corrected transposition of the great arteries)

Significant	Transposition of the great vessels, tetralogy of Fallot, large ventricular septal defect, coarctation of the aorta, aortopulmonary window, critical aortic or pulmonary stenosis, partial atrioventricular septal defect, total anomalous pulmonary venous connection, and tricuspid valve dysplasia (no Ebstein's anomaly)

Minor	Small ventricular septal defect and less severe aortic or pulmonary stenosis

Others	Dysrhythmias, cardiomyopathies, secondary dextrocardia/levocardia, pulmonary sequestration, and restrictive ductus arteriosus

*This classification was adapted from Hunter et al. [17] and Wren et al. [18].

**Table 2:** Baseline characteristics of the patients.

	Normal heart (*n* = 244)	Abnormal heart (*n* = 27)	Total (*n* = 271)	*P*
Maternal age at echo				0.17^a^
≥35 years old, *n*° (%)	103 (42%)	8 (29.6%)	111 (40.9%)	
Gestation age at echo (weeks), mean (standard deviation)	27.8 (±4.6)	28.9 (±4.6)	27.8 (±4.6)	0.84^b^
Twin pregnancy, *n*° (%)	7 (2.8%)	1 (3.7%)	8 (2.9%)	0.58^c^
Race, *n*° (%)				0.07^c^
White	78 (45.4%)	8 (36.4%)	87 (44.6%)	
Black	24 (13.9%)	1 (4.6%)	25 (12.8%)	
Mixed	68 (39.5%)	11 (50%)	79 (40%)	
Asian	2 (1.2%)	2 (9%)	4 (2%)	
Indication of screening*, *n*° (%)				<0.001^c^
Maternal cause	180 (73%)	3 (11%)	183 (67%)	
Fetus cause	66 (27%)	24 (89%)	90 (33%)	

^a^Chi Pearson test. ^b^Mann-Whitney test. ^c^Exact Fisher test. *Some cases had one more indication.

**Table 3:** Reasons for fetal heart screening and frequency of congenital heart disease.

Reasons for screening*	Normal heart (*n* = 244)	Abnormal heart (*n* = 27)	Total (*n* = 271)	CHD among referral reason
Maternal indications	180	3	183 (67%)	3/183 (1.6%)
Advanced maternal age (≥35 yo)	51	1	52 (19%)	1/52 (1.9%)
History of CHD	26	2	28 (10%)	2/28 (7.1%)
Preexisting metabolic disease	81	1	82 (30%)	1/82 (1.2%)
Infections	11	—	11 (4%)	—
Teratogen exposure	4	—	4 (1.4%)	—
Others	7	—	7 (2.5%)	—
Fetal indications	66	24	90 (33%)	24/90 (26.7%)
Cardiac abnormality/dysrhythmia	10	19	29 (10.7%)	19/29 (65.5%)
Extracardiac abnormality	39	7	46 (17%)	7/46 (15%)
Central nervous system	25	3	28 (10.3%)	3/28 (10.7%)
Abdominal wall defect	2	1	3 (1%)	1/3 (33%)
Diaphragmatic hernia	—	1	1 (0.4%)	—
Functional renal agenesis	6	1	7 (2.5%)	1/7 (14%)
Others	6	1	7 (2%)	—
Intrauterine growth restriction	3	—	3 (1%)	—
Others	14	—	14 (5.1%)	—

*Some cases had one more indication. CDH: congenital heart disease; yo: years old.

**Table 4:** Congenital heart disease recognised prenatally by classification system of fetal heart diseases according to complexity of the heart anatomical abnormalities.

Cardiac abnormality	Frequency (*n* = 27)
Complex	13 (48.1%)
Heterotaxy or atrial isomerism	1
Hypoplastic left heart syndrome	6
Tricuspid atresia	1
Ebstein's anomaly	2
Truncus arteriosus	1
Complete atrioventricular septal defect	1
Double outlet of right ventricle	1
Significant	5 (18.5%)
Tetralogy of Fallot	2
Large ventricular septal defect	1
Tricuspid valve dysplasia	1
Critical pulmonary stenosis	1
Minor	2 (7.4%)
Small ventricular septal defect	2
Others	7 (26%)
Dysrhythmias	1
Cardiomyopathies	2
Pulmonary sequestration	1
Secondary dextrocardia/levocardia	2
Restrictive ductus arteriosus	1
